# A reference genome for the nectar-robbing Black-throated Flowerpiercer (*Diglossa brunneiventris*)

**DOI:** 10.1093/g3journal/jkab271

**Published:** 2021-08-02

**Authors:** Anna E Hiller, Robb T Brumfield, Brant C Faircloth

**Affiliations:** Museum of Natural Science and Department of Biological Sciences, Louisiana State University, Baton Rouge, LA 70803, USA

**Keywords:** Thraupidae, *Diglossa brunneiventris*, Flowerpiercers, long-read sequencing, genome assembly

## Abstract

Black-throated Flowerpiercers (*Diglossa brunneiventris*) are one species representing a phenotypically specialized group of tanagers (Thraupidae) that have hooked bills which allow them to feed by stealing nectar from the base of flowers. Members of the genus are widely distributed in montane regions from Mexico to northern Argentina, and previous studies of *Diglossa* have focused on their systematics, phylogenetics, and interesting natural history. Despite numerous studies of species within the genus, no genome assembly exists to represent these nectivorous tanagers. We described the assembly of a genome sequence representing a museum-vouchered, wild, female *D. brunneiventris* collected in Peru. By combining Pacific Biosciences Sequel long-read technology with 10× linked-read and reference-based scaffolding, we produced a 1.08 Gbp pseudochromosomal assembly including 600 scaffolds with a scaffold N50 of 67.3 Mbp, a scaffold L50 of 6, and a BUSCO completeness score of 95%. This new assembly improves representation of the diverse species that comprise the tanagers, improves on scaffold lengths and contiguity when compared to existing genomic resources for tanagers, and provides another avenue of research into the genetic basis of adaptations common to a nectivorous lifestyle among vertebrates.

## Introduction

**Figure 1:**
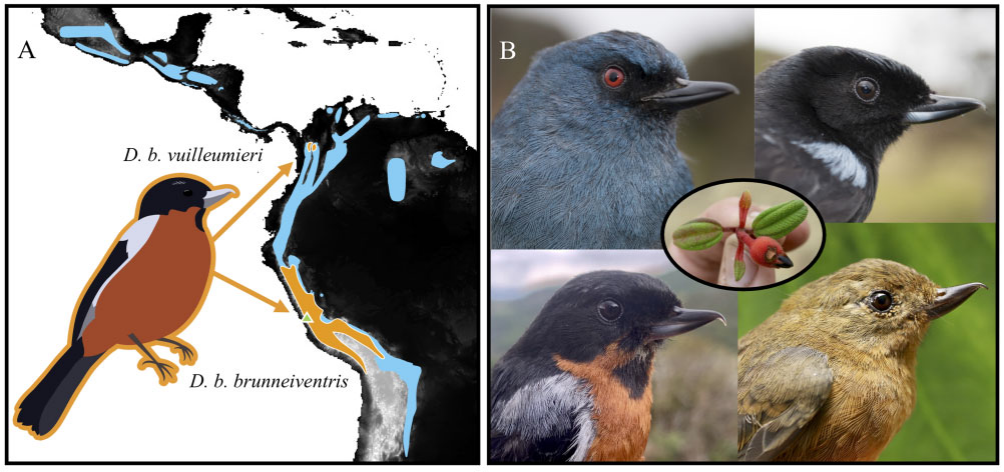
(A) A range map for the *Diglossa* flowerpiercers with the distribution of the genus shown in blue, and a range map for both subspecies of *D. brunneiventris* shown in orange accompanied by an illustration of the focal species (adapted from BirdLife International and Handbook of the Birds of the World 2019). Locality for the reference genome individual *D. brunneiventris* is indicated by the green triangle. (B) Examples of the phenotypes observed among *Diglossa* flowerpiercers. Top left: *D. caerulescens*, top right: *D. humeralis* (photos by Diego Cueva), bottom left: *D. brunneiventris*, bottom right: *D. albilatera* female (photos by Anna Hiller). The central photo shows a flower of *Brachyotum* sp. with a “pierced” corolla (photo by Anna Hiller).

Flowerpiercers in the genus *Diglossa* are a phenotypically specialized group of tanagers known for their unique adaptation for feeding—a hooked bill that they use to pierce the base of flowers—and an associated behavior called nectar-robbing (Inouye 1983; Bock 1985; Mauck and Burns 2009). The 18 species of flowerpiercer are phenotypically diverse and show variation in body size, bill shape, plumage color and patterning, and eye color (Zimmer 1929; Isler and Isler 1987; del Hoyo *et al.* 1992; Mauck and Burns 2009; Figure 1). *Diglossa* species vary in their degree of sexual dichromatism, and there are several examples of phenotypic convergence in the group (*e.g.*, “leapfrog” variation where disjunct populations are phenotypically similar; Zimmer 1929; Vuilleumier 1969; Remsen 1984). *Diglossa* are widely distributed in montane regions from Central Mexico to northern Argentina with peak species diversity in the equatorial Andes (Vuilleumier 1969; Isler and Isler 1987; del Hoyo *et al.* 1992; Figure 1). Two flowerpiercers, *D. gloriossisima and D. venezuelensis*, are species of conservation concern (del Hoyo *et al.* 1992; IUCN 2020). 

Previous studies of *Diglossa* have focused on their systematics (Zimmer 1929; Zimmer *et al.* 1952; Bock 1985; Graves 1990), phylogenetics (Hackett 1995; Mauck and Burns 2009), biogeography (Chapman 1926; Vuilleumier 1969; Graves 1982, 1985, 1988; Navas Berdugo 2008; Cuervo 2013; Gutiérrez-Zuluaga *et al.* 2021), natural history (Lyon and Chadek 1971; Colwell *et al.* 1974; Graves 1982; Stiles *et al.* 1992; Rojas-Nossa 2007; Solano-Ugalde and Brinkhuizen 2012; Vaicenbacher *et al.* 2014; Hazlehurst and Karubian 2016), and relationships to other taxonomic groups within the Thraupidae (Burns 1997; Burns *et al.* 2003, 2014), one of the most species-rich bird families. Despite the ecological and evolutionary uniqueness of *Diglossa*, there are no genome assemblies available for these nectivorous tanagers, and only four genome assemblies (Supplementary Table S1) represent the 98 genera and 371 named species within the tanager family (Dickinson and Christidis 2014).

Here, we announce the assembly of a genome from a wild caught, museum-vouchered (Buckner *et al.* 2021), female Black-throated Flowerpiercer (*Diglossa* *brunneiventris*) collected in the Department of Lima, Peru. We selected this species among other *Diglossa* because of its role in foundational studies of biogeography (Chapman 1926; Hazzi *et al.* 2018), ecogeographic variation in plumage and body size (Remsen 1984; Graves 1991), secondary contact and hybridization (Graves 1982), and the genetics of high-altitude blood physiology (Natarajan *et al.* 2016). A reference assembly for the Black-throated Flowerpiercer will enable related studies on the role of genetic architecture in reproductive isolation and adaptation, the genetics of color and patterning in birds, and the impact of hybridization on speciation processes.

## Materials and methods

### Specimen collection and high-molecular-weight DNA extraction

We collected blood and other tissues from a wild, female bird captured 6.3 km east-southeast of San Mateo, Department of Lima, Peru (11.775 S, 76.245 W, 3900 m) during October 2018 under permits from the Servicio Nacional Forestal y de Fauna Silvestre (SERFOR; collecting permits 203-2015-SERFOR-DGGSPFFS, 222-2015-SERFOR-DGGSPFFS, export permit 003336 SERFOR) and following LSU’s Institutional Animal Care and Use Committee (IACUC) protocol number 18-054. We selected this population because it was far from any potential zone of secondary contact and/or hybridization with other *Diglossa* species (Graves 1982). We stored blood in a cryotube with 95% ethanol, placed the blood in liquid nitrogen, and flash froze all remaining tissues (liver, pectoral and cardiac muscle, ovary, eyes, and brain) in liquid nitrogen. Following tissue collection, we prepared a specimen for the LSU Museum of Natural Science (LSUMNS) Collection of Birds (LSUMZ 227748), and we deposited tissue samples from this specimen into the LSUMNS Collection of Genetic Resources (LSUMZ B-95988) as well as the tissue collection of the Centro de Ornitología y Biodiversidad (CORBIDI) in Lima, Peru.

To minimize contaminating mitochondrial sequences, we extracted high-molecular weight (HMW) DNA from the blood we collected using a modified phenol-chloroform protocol (Supplementary File S1). After extraction, we performed a bead cleanup using an inexpensive alternative to commercial SPRI reagents (Glenn *et al.* 2019) to remove remaining impurities and short DNA fragments. We quantified the extracted DNA using a Qubit fluorometer (Thermo Fisher Scientific, Inc.) with the broad spectrum kit, and we determined the size distribution of the DNA by analyzing a 100 ng/µL aliquot using an Agilent TapeStation and the Genomic DNA ScreenTape (Kansas University Medical Center, Kansas City, KS, USA). We shipped two aliquots totaling 8.5 µg HMW DNA on dry ice to the Georgia Genomics Facility (GGF; Athens, GA, USA) for library preparation.

### Library preparation and sequencing

We were interested in comparing an assembly from Pacific Biosciences (hereafter PacBio) Sequel long-reads technology to an assembly from 10× Genomics Chromium linked-reads technology, so we had the GGF staff prepare: (1) a single 10× linked-read library from <0.5 µg HMW DNA using the Chromium Genome Library Kit (v2) and Chromium Genome Chip Kit (v2), and (2) a single SMRTbell library from 5.0 µg input DNA using the SMRTbell Express Template Prep Kit (v1.0) following the guidelines for preparing 20 kb templates.

After preparing the 10× linked-read library, GGF staff determined the insert size of the library using an Agilent Fragment Analyzer and quantified the library using a Qubit Fluorometer. We sequenced the library using a partial Illumina HiSeq X (Novogene, Inc.) lane, targeting 250 million paired-end, 150 bp reads to achieve ∼56X coverage assuming a final assembly of ∼1.1 Gbp (Feng *et al.* 2020). The libraries sharing the remainder of the HiSeq X lane had nonoverlapping 10× barcodes.

After preparing the SMRTbell Express 20 kb library and performing quality control steps following the manufacturer’s instructions, GGF staff sequenced an aliquot of the resulting library using one SMRT Cell 1M (v3) on a PacBio Sequel System with software version 6.0.x and chemistry version 3.0 (movie time: 600 m; immobilization time: 120 m). Because the initial SMRT Cell performed nominally, we had GGF staff sequence remaining aliquots of the same library on two additional SMRT Cells using the same run parameters in order to approach ∼30X coverage.

### Genome assembly

We assembled all 10× linked-read sequencing data (–maxreads=“all”) with the Supernova (v2.2.1) software on a compute node using 24 compute cores and 745 GB RAM. After successfully running the Supernova pipeline, we output all versions of the assembly, although subsequent comparisons were performed using the pseudohaploid version of the assembly. Specifically, we computed assembly contiguity statistics with Quast (Gurevich *et al.* 2013; v5.0.2) and estimated assembly completeness using BUSCO (Seppey et al. 2019; v4.0.6). Because the linked-read, 10× sequences can also be used for other purposes (like assembly polishing) once the internal barcodes are trimmed, we processed the raw, linked-read FASTQ files using the “basic” analysis of the 10× Genomics Long Ranger analysis pipeline (v2.2.2) to output reads that were trimmed of their 10× barcodes.

Prior to assembling the PacBio Sequel long-read data, we converted the subread BAM files to FASTQ format using bam2fastq (v1.3.0) from the PacBio BAM2fastx package (https://github.com/PacificBiosciences/bam2fastx; accessed 2021 August 2). We assembled the FASTQ files using canu (Koren *et al.* 2017; v1.8, r9465) on a 48-core compute node with 1.5 TB RAM and default options, other than setting the genome size parameter to 1.1 Gbp. Following this initial round of contig assembly, we computed contiguity statistics with Quast and estimated completeness using BUSCO, so that we could compare both metrics to the 10× linked-read assembly.

Based on the results of this initial comparison, we decided to move forward with the PacBio assembly. We aligned the short-read data, trimmed of 10× barcodes, to the PacBio contigs using bwa-mem (Li 2013; v0.7.17-r1188) and samtools (Li *et al.* 2009; v1.9) with default options, and we polished the contigs with Pilon (Walker *et al.* 2014; v1.23) using default options on a 48-core compute node with 1.5 TB RAM. We performed an initial round of misassembly correction using tigmint (v1.1.2) followed by contig scaffolding using arks+links (Warren *et al.* 2015; Coombe *et al.* 2018; Jackman *et al.* 2018; arks v1.0.4) with the Long Ranger-processed 10× reads and a file of barcode multiplicities we generated using the “calcBarcodeMultiplicities.pl” helper script from the arks software package. After the first round of scaffolding, we cleaned the contig names using custom Python code and removed contigs <1000 bp in length using “faFilter” from the UCSC Genome Browser software package (Kent *et al.* 2002, v25-Mar-2014-Kent-Source-LINUX-x86_64). Then, we computed contiguity statistics with Quast and estimated completeness using BUSCO. The BUSCO results showed a moderate level of duplication among BUSCOs, suggesting that the assembly contained a number of haplotigs. In order to identify and remove these, we modeled repeats in the scaffolded assembly using RepeatModeler2 (Flynn *et al.* 2020, v2.0.1) and RMBlast (v2.9.0), and we identified repeats using RepeatMasker (Smit *et al.* 2019; v4.1.0) and RMBlast (v2.9.0) with the sensitive option (-s) and output repeats in GFF format (-gff). We converted the GFF file of repeat locations to BED using awk (GNU Software Foundation 2020), and we removed putative haplotigs from the assembly using purge_haplotigs (Roach *et al.* 2018; v1.1.1) with this BED-formatted file of repeat locations. After haplotig purging, we computed contiguity statistics with Quast and estimated assembly completeness using BUSCO.

We converted the gaps in the arks-scaffolded assembly from 100 to 50 bp using custom Python code, and we performed a round of reference-based scaffolding using ragtag (Alonge *et al.* 2019; v1.0.0) and the *Camarhyncus parvulus* assembly (GCF_901933205.1; last common ancestor ∼12–10 Ma; Barker *et al.* 2013). We filtered the *Camarhyncus* assembly to contain only chromosomes, and we scaffolded using ragtag with default options (setting gaps to 100 bp so that pseudochromosomal scaffolds can be easily identified and/or split). We renamed the pseudochromosomal scaffolds to reflect the reference genome used to scaffold them using custom Python code, uppercased the entire assembly, and ran the unmasked, ragtag-scaffolded assembly through RepeatMasker using the repeat models created above. We computed a final round of contiguity statistics with Quast and estimated completeness using BUSCO.

## Results and discussion

Illumina sequencing of the 10× linked-read library generated 290,045,347 read pairs. Supernova software trimmed reads to a mean length of 139 bp and estimated raw coverage as 60X (41X “effective coverage”). After completion of the Supernova assembly pipeline, the pseudohaploid assembly included 26,480 contigs with an N50 of 6.1 Mbp (L50 50; Table 1). Total assembly length was 1.10 Gbp. BUSCO scores suggested the 10× assembly was reasonably complete (Table 2), although the Supernova assembly was less contiguous than any version of the canu assembly (Table 1; see below).

**Table 1:** Quast summary statistics for different versions of the *Diglossa brunneiventris* assemblies

Assembly	Supernova	Canu	Canu+*pol*	Canu+*pol+scaf1*	Canu+*pol+ scaf1+purg*	Canu+*pol+ scaf1+purg+scaf2*
# contigs (≥0 bp)	26,480	3087	3087	2960	1328	601
# contigs (≥1000 bp)	26,480	3087	3087	2960	1328	601
# contigs (≥5000 bp)	5499	2971	2971	2762	1260	555
# contigs (≥10,000 bp)	1967	2880	2880	2619	1197	513
# contigs (≥25,000 bp)	803	2628	2628	2344	1049	426
# contigs (≥50,000 bp)	596	1960	1962	1714	829	285
Total length (≥0 bp)	1.11E+09	1.17E+09	1.17E+09	1.17E+09	1.08E+09	1.08E+09
Total length (≥1000 bp)	1.11E+09	1.17E+09	1.17E+09	1.17E+09	1.08E+09	1.08E+09
Total length (≥5000 bp)	1.06E+09	1.17E+09	1.17E+09	1.17E+09	1.08E+09	1.08E+09
Total length (≥10,000 bp)	1.04E+09	1.17E+09	1.17E+09	1.17E+09	1.08E+09	1.08E+09
Total length (≥25,000 bp)	1.02E+09	1.17E+09	1.17E+09	1.17E+09	1.08E+09	1.08E+09
Total length (≥50,000 bp)	1.02E+09	1.14E+09	1.14E+09	1.14E+09	1.07E+09	1.07E+09
Largest contig	22,027,751	28,875,242	28,916,091	30,446,970	30,446,970	155,774,234
N50	6,056,454	6,979,468	6,984,183	9,220,893	10,495,952	67,281,049
N75	1,734,222	937,141	934,919	1,297,763	2,420,771	22,837,129
L50	50	46	46	37	32	6
L75	129	164	165	123	83	12
# N's per 100 kbp	1593	—	—	2	2	8

Canu assemblies are named according to the steps used to produce each (*pol*: polished; *scaf1*: arks scaffolded; *purg*: haplotig purged; *scaf2*: ragtag scaffolded).

**Table 2:** BUSCO statistics for different versions of the *Diglossa brunneiventris* assemblies

Assembly	Supernova		Canu		Canu+pol		Canu+pol+scaf		Canu+pol+scaf+purg		Canu+pol+scaf+purg+scaf	
Complete BUSCOs (C)	237	93%	238	93%	242	95%	241	94.5%	242	95%	241	95%
Complete and single-copy BUSCOs (S)	234	92%	228	89%	227	89%	226	88.6%	240	94%	239	94%
Complete and duplicated BUSCOs (D)	3	1%	10	4%	15	6%	15	5.9%	2	1%	2	1%
Fragmented BUSCOs (F)	11	4%	6	2%	2	1%	3	1.2%	3	1%	4	2%
Missing BUSCOs (M)	7	3%	11	4%	11	4%	11	4.3%	10	4%	10	4%
Total BUSCO groups searched	255		255		255		255		255		255	

Canu assemblies are named according to the steps used to produce each (*pol*: polished; *scaf1*: arks scaffolded; *purg*: haplotig purged; *scaf2*: ragtag scaffolded).

PacBio sequencing of the SMRTbell Express 20 kb library across three SMRT Cells produced 1.6 million reads having a mean value for longest subread of 20,319 bp (95 CI ± 1980 bp) and a mean value for longest subread N50 of 33,144 (95 CI ± 1369). Sequencing produced a total of 32.7 Gbp of data. Canu produced an assembly including 3087 contigs having an N50 of 6.9 Mbp (L50 46), a total length of 1.17 Gbp, and BUSCO scores that indicated a reasonable level of completeness with a moderate level of duplicated BUSCOs (Table 2). Polishing improved the detection of several missing BUSCOs, while it also increased the number of duplicated BUSCOs (Table 2). Assembly correction and scaffolding with tigmint, arks, and links increased the N50 by 3 Mbp (Table 1) with negligible effects on assembly completeness (Table 2). Visual analysis of the coverage histogram produced by purge_haplotigs suggested that appropriate coverage cutoffs were 5 (low read depth cutoff), 15 (the point between haploid and diploid peaks), and 190, and haplotig purging considering repeat regions removed a number of low coverage contigs (*n* = 1632) from the assembly and reduced the number of duplicated BUSCOs to a low level (∼1%) with minor effects (∼0.4% reduction) on the number of complete and single copy BUSCOs detected. Removal of putative haplotigs reduced the assembly length from 1.17 to 1.08 Gbp (Table 1). Reference-guided scaffolding using the *C.* *parvulus* assembly increased assembly contiguity to chromosome-level while retaining BUSCO scores similar to previous assembly versions (Tables 1 and 2).

The highly contiguous assembly we produced for *Diglossa* contributes to the growing number of assemblies representing the incredible diversity of this important avian family, enabling future studies of how, when, and where tanagers diversified. More broadly, sequencing and assembling a genome from a nectivorous tanager adds to the collection of obligate nectivores sequenced among the vertebrate Tree of Life (Feng *et al.* 2020; Supplementary Table S2), providing another avenue of research into the genetic basis of adaptations common to a nectivorous lifestyle, including: sweet taste perception (*e.g.*, Baldwin *et al.* 2014; Toda *et al.* 2021), sugar metabolism (*e.g.*, Schondube and Del Rio 2004; Workman *et al.* 2018), spatial memory of floral resources (*e.g.*, Araya-Salas *et al*., 2018), frilled channeled tongues (*e.g.*, Rico-Guevara *et al.* 2019), and whether such adaptations have evolved through similar or different molecular mechanisms.

## Data availability

Raw sequencing data are available from NCBI (PRJNA629984). 10× Supernova Assemblies are available from https://doi.org/10.5281/zenodo.4439775, while the pseudochromosomal, PacBio assembly has been deposited at DDBJ/ENA/GenBank under the accession JAFCGX000000000. The version described in this study is version JAFCGX010000000. Two supplemental tables describe available tanager genome assemblies (Supplementary Table S1), and assemblies for other vertebrate organisms that are obligate nectivores (Supplementary Table S2), one supplemental file describes our DNA extraction protocol (Supplementary File S1), a second supplemental file describes all processing steps and commands (Supplementary File S2), and a final set of supplemental files provides the RepeatModeler and RepeatMasker output (Supplementary File S3). Supplementary tables and files are available from FigShare: https://doi.org/10.25387/g3.15030714. Samples were collected under collecting permits 203-2015-SERFOR-DGGSPFFS and 222-2015-SERFOR-DGGSPFFS, exported under permit 003336 SERFOR, and collected following LSU IACUC protocol 18-054.
